# Comparative ^1^H NMR-Based Metabolomics of Traditional Landrace and Disease-Resistant Chili Peppers (*Capsicum annuum* L.)

**DOI:** 10.3390/foods13131966

**Published:** 2024-06-21

**Authors:** Gi-Un Seong, Dae-Yong Yun, Dong-Hyeok Shin, Jeong-Seok Cho, Gyuseok Lee, Jeong Hee Choi, Kee-Jai Park, Kyung-Hyung Ku, Jeong-Ho Lim

**Affiliations:** 1 Food Safety and Distribution Research Group, Korea Food Research Institute, Wanju-gun 55365, Republic of Korea; s.giun@kfri.re.kr (G.-U.S.); ydy0401@kfri.re.kr (D.-Y.Y.); s.donghyeok@kfri.re.kr (D.-H.S.); jscho@kfri.re.kr (J.-S.C.); choijh@kfri.re.kr (J.H.C.); jake@kfri.re.kr (K.-J.P.); khku@kfri.re.kr (K.-H.K.); 2 Smart Food Manufacturing Project Group, Korea Food Research Institute, Wanju-gun 55365, Republic of Korea; gslee@kfri.re.kr

**Keywords:** chili pepper, *Capsicum annuum*, metabolomics, nuclear magnetic resonance, disease-resistant cultivars, multivariate analysis, landrace, Subicho

## Abstract

Chili peppers (*Capsicum annuum* L.) are economically valuable crops belonging to the Solanaceae family and are popular worldwide because of their unique spiciness and flavor. In this study, differences in the metabolomes of landrace (Subicho) and disease-resistant pepper cultivars (Bulkala and Kaltanbaksa) widely grown in Korea are investigated using a ^1^H NMR-based metabolomics approach. Specific metabolites were abundant in the pericarp (GABA, fructose, and glutamine) and placenta (glucose, asparagine, arginine, and capsaicin), highlighting the distinct physiological and functional roles of these components. Both the pericarp and placenta of disease-resistant pepper cultivars contained higher levels of sucrose and hexoses and lower levels of alanine, proline, and threonine than the traditional landrace cultivar. These metabolic differences are linked to enhanced stress tolerance and the activation of defense pathways, imbuing these cultivars with improved resistance characteristics. The present study provides fundamental insights into the metabolic basis of disease resistance in chili peppers, emphasizing the importance of multi-resistant varieties to ensure sustainable agriculture and food security. These resistant varieties ensure a stable supply of high-quality peppers, contributing to safer and more sustainable food production systems.

## 1. Introduction

The chili pepper (*Capsicum annuum* L.) is an important crop of the nightshade family Solanaceae. During the Columbian Exchange, this crop was introduced into various Asian countries and became dispersed worldwide. Global cuisine regularly employs immature and mature chili peppers owing to their pungent flavor and high nutritional value. Five species within the Capsicum genus have been domesticated, namely *C. annuum*, *C. baccatum*, *C. chinense*, *C. frutescens*, and *C. pubescens* [1]. The most commonly cultivated pepper groups are the bell, sweet, and hot peppers. In addition, Korean chili peppers are unique among global hot pepper species (Hungarian, Sichuan, Italian, and Greek peppers) owing to their moderate spiciness and distinct sweet flavor [2]. Chili peppers also have various medicinal uses owing to their high levels of capsaicin, which has significant pharmacological properties. Capsaicin is used as an analgesic in topical medications for pain relief and in weight loss supplements to increase metabolism and reduce appetite [3]. Chili peppers are also rich in vitamins A and C, which contribute to their antioxidant properties, potentially reduce the risk of chronic diseases, and support immune function [4]. Chili pepper cultivars, including landraces, are superior varieties acclimatized to specific environmental conditions and meticulously selected by farmers over an extended period of time. The Subicho species is a representative species prized for its unique appearance, taste, and high milling rate [5]. Although these cultivars have advantages in terms of their quality and pricing, they are typically produced in low yields and are highly susceptible to disease. Indeed, chili peppers are affected by various diseases and have a high incidence of pathogens and viruses. Anthracnose is a particularly prevalent disease that causes significant damage to chili peppers, resulting in post-harvest yield losses ranging from 12 to 50% [6]. Furthermore, viral infections such as those caused by the tomato spotted wilt virus (TSWV), the cucumber mosaic virus (CMV), and the broad bean wilt virus (BBWV2) cause a wide range of symptoms that exacerbate the difficulties encountered by chili pepper cultivators [7]. Recent advances in breeding programs designed to tackle these challenges have produced chili pepper cultivars that exhibit both primary resistance and resilience to anthracnose. This was achieved using a multi-complex disease-resistant cultivar [8].

Metabolomics explores biological systems by monitoring and analyzing the metabolomes of living organisms. Proton nuclear magnetic resonance (^1^H NMR)-based metabolomics is commonly employed to determine unknown metabolite structures owing to its simple sample preparation techniques and rapid detection of diverse primary and secondary metabolites [9]. Metabolomics combines experimental design, sample preparation, chemical analysis, data processing, and bioinformatics to gain a deeper understanding of the chemical and phenotypic variations among plants [10]. This understanding extends to a range of conditions, such as genetic modification, different origins, environmental and pathogenic stresses, and varying growth conditions [11,12]. In addition, the combination of metabolic profiling with multivariate analysis can identify differences in the metabolite levels between samples, thereby enabling species classification.

In the current study, the metabolic diversity of traditional and disease-resistant chili pepper cultivars is examined by analyzing the pericarp, placenta, and seed components of each cultivar using advanced metabolomics techniques such as clustering comparison using SIMCA and pairwise OPLS-DA. Our findings improve our understanding of the factors influencing disease resistance and quality in chili peppers and will therefore contribute to advancements in their cultivation and breeding. Furthermore, the application of this research in the food industry will ensure a stable supply of high-quality peppers, promoting safer and more sustainable food production.

## 2. Materials and Methods

### 2.1. Plant Source and Sample Preparation

*Capsicum annuum* L. chili peppers, a domesticated species of the plant genus Capsicum in the family Solanaceae, were grown at the Yeong-yang Pepper Experiment Station (Yeongyang, Republic of Korea) following standard agronomic practices. All three cultivars used, i.e., the Subicho, Bulkala, and Kaltanbaksa cultivars, belong to this classification. The seeds were sown in mid-February, with seedlings grown for 70–80 days before being transplanted in early May. The peppers were harvested in early August, with optimal growth temperatures maintained between 20 and 30 °C until the 2022 harvest. Subicho, a landrace variety, has been cultivated in the Yeongyang region of Korea since 1950 and has consistently been well-received by consumers [13]. In the 2010s, the Bulkala and Kaltanbaksa cultivars gained popularity among growers owing to their enhanced disease-resistance characteristics [14]. The Bulkala cultivar demonstrates a high resistance to the TSWV, Phytophthora blight, and the CMV, while the Kaltanbaksa cultivar is a noteworthy multi-disease-resistant chili pepper cultivar that exhibits resistance to TSWV, Phytophthora blight, CMV, and anthracnose.

Fully ripened chili pepper plants grown in open fields were examined, with a focus on the pericarp, placenta, and seeds. The collected chili peppers were immediately frozen in liquid nitrogen and stored at −80 °C until required for extraction or processing. The freeze-dried chili peppers were manually separated into their respective parts using a sterile scalpel. These chili pepper components were ground in liquid nitrogen using an analytical grinding mill (model A 11, IKA Works Inc., Freiburg im Breisgau, Germany). The ground samples were then transferred to Eppendorf tubes, stored at −80 °C for 24 h in a freezer, and freeze-dried for 48 h. Extracts of each freeze-dried sample were prepared and analyzed using ^1^H NMR spectroscopy.

### 2.2. ^1^H NMR Spectroscopic Analysis of Chili Pepper Extracts

The extraction of metabolites from the chili pepper sample components was performed in accordance with the protocol described by Yun et al. [15]. Deuterated methanol-*d*4 (CD_3_OD, 99.8 atom% D), potassium dihydrogen phosphate (KH_2_PO_4_), sodium deuterium oxide (NaOD), and deuterium oxide (D_2_O, 99.9% D) were supplied by Sigma-Aldrich (St. Louis, MO, USA). A buffer was prepared by adding KH_2_PO_4_ (1.232 g) to D_2_O (37.5 mL). The pH of this buffer was adjusted to pH 6 using NaOD, and CD_3_OD (62.5 mL) was added. Each freeze-dried sample (20 mg) was dissolved in the buffer (1 mL) in an Eppendorf tube. The resulting mixture was sonicated for 20 min at 25 °C to extract the metabolites from the chili pepper samples, and centrifugation was then performed at 13,000 rpm and 5 °C for 20 min. The supernatant from each chili pepper extract (550 μL) was transferred to an NMR tube. A total of 90 samples were analyzed, comprising 10 samples from each edible component (pericarp, placenta, seeds) of the three chili pepper cultivars (Subicho, Bulkala, Kaltanbaksa). Additionally, a quality control (QC) sample was prepared by pooling equal volumes of all chili extracts for NMR analysis [16]. The CD_3_OD present in the supernatant was used as the field frequency lock, and the methyl group signal (methanol-*d*4) was employed as a chemical shift reference (^1^H, δ 3.324). The ^1^H NMR spectra were acquired using a Bruker Avance 800 spectrometer (Bruker Biospin, Rheinstetten, Germany) operating at 800 MHz and 298 K. The spectrometer was equipped with a cryogenic triple-resonance probe and a Bruker automatic injector. One-dimensional-^1^H-nuclear Overhauser effect spectroscopy (1D-NOESY) was performed using a pulse sequence from the Bruker library (noesygppr1d). Two-dimensional (2D) ^1^H–^1^H total correlation spectroscopy (TOCSY) and ^1^H–^13^C heteronuclear single-quantum correlation (HSQC) spectra were acquired using dipsi2esgpph and hsqcetgpsisp2 pulse sequences, respectively, from the Bruker library. Signals in the spectra of representative QC samples were assigned based on the corresponding two-dimensional TOCSY and HSQC spectra.

### 2.3. NMR Data Processing and Multivariate Statistical Analysis

All NMR spectra were manually calibrated using CD_3_OD (^1^H, d 3.324) and corrected for phase and baseline distortions in Topspin (TopSpin 4.3.0, Bruker Biospin, Rheinstetten, Germany). The calibrated and corrected spectra were then imported into MATLAB (R2014a; The Mathworks Inc., Natick, MA, USA) and further aligned using the *i*coshift and correlation optimized warping (COW) methods [17,18]. The spectra were normalized using total area and quotient probabilistic methods to avoid dilution effects. Multivariate statistical analysis (MVA) of full resolution ^1^H NMR spectra was performed without spectrum bucketing or binning, excluding unnecessary regions from d 0 to 0.5 ppm, d 3.31 to 3.33 ppm, and d 9.6 to 10 ppm. MVA of the integral datasets of assigned metabolites was further performed and visualized using orthogonal projection to latent structure discriminant analysis (OPLS-DA) score plots. The resulting datasets were imported into SIMCA (version 18.0; Sartorius Stedim Biotech, Umeå, Sweden) and MVA was performed using a mean-centered scaling method. Principal component analysis (PCA), an unsupervised pattern recognition method, was initially conducted to investigate the intrinsic variation in the dataset. OPLS-DA was employed as a supervised pattern recognition method to extract information from the discriminant compounds contained in the dataset. Notably, OPLS-DA aids in removing systematic variations from an input dataset X (compounds or metabolites) that is not correlated with the response set Y (discriminant classes). Hotelling’s *T*-squared distribution (*T*^2^; 95%) was calculated in SIMCA to identify strong outliers within each sample, ensuring that all available data were within the 95% confidence interval. The OPLS-DA models included a 7-fold cross-validation method and a permutation test with 200 iterations. To enhance the interpretation of results and identify metabolites contributing to metabolic discrimination between the two classes, OPLS loading or coefficient plots were generated with color-coded correlation coefficients for each data point using MATLAB (R2014a) with scripts developed at Imperial College London. The model quality was assessed using the R^2^X, R^2^Y, and Q^2^ values, where R^2^X is the proportion of variance in the data explained by the models, indicating the goodness of fit, R^2^Y is the extent to which the model explains the variance in the dependent variable, and Q^2^ is the proportion of the variance in the data predicted by the model. The relative quantification of the metabolites was performed using the integral area of each metabolite’s corresponding peak in the ^1^H NMR spectra. The signal ranges specified in Table S1 were used, and those that overlapped with other metabolites were excluded from the analysis.

### 2.4. Statistical Analysis

The results are presented as the mean ± standard deviation of 10 samples of each edible component of the three chili pepper varieties. The statistical significance was determined using one-way ANOVA and Duncan’s multiple range test. A *p*-value of <0.05 was considered statistically significant. The paired Student *t*-test was used to validate the significance of the metabolite differences observed in the OPLS coefficient or the loading plot for pairwise comparison.

## 3. Results and Discussion

### 3.1. Identification of Metabolites in the Chili Pepper Extracts by 1D and 2D NMR Spectroscopy

**Figure 1:**
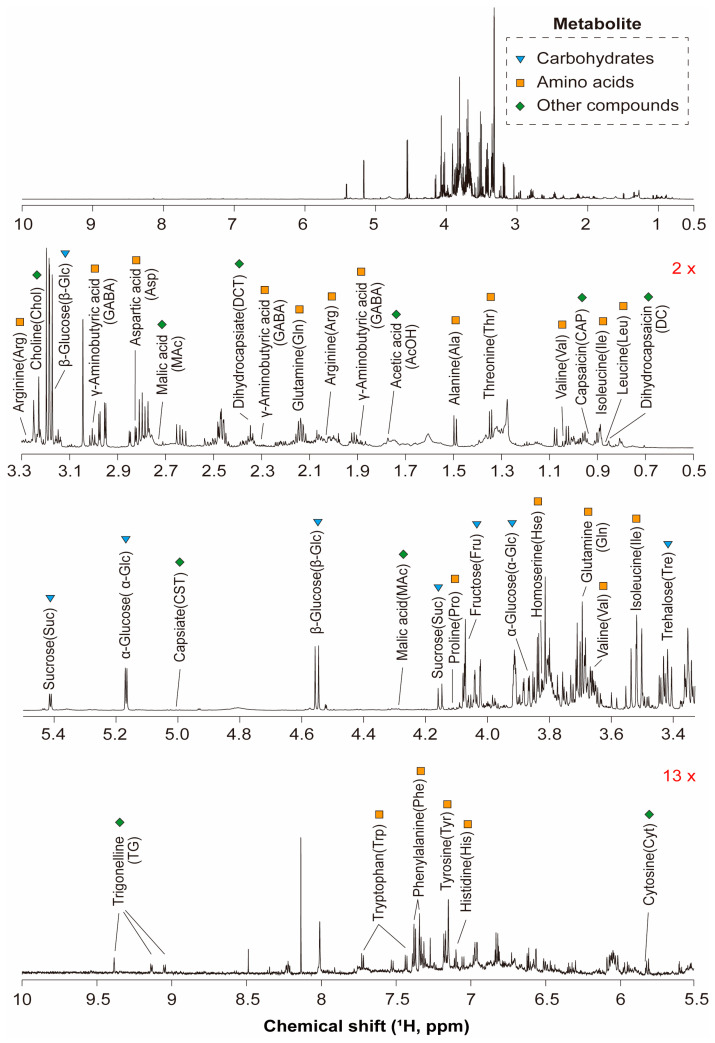
Representative ^1^H NMR spectrum (800 MHz) of the pooled QC sample prepared by pooling equal volumes of all chili extracts.

The one-dimensional (1D) ^1^H NMR spectra of the pericarp, placenta, and seed components of the chili pepper samples comprised three main chemical shift regions incorporating a wide range of metabolites, including carbohydrates, amino acids, organic acids, nucleotides, and other compounds (Figure 1). The signals corresponding to each metabolite were confirmed by 2D NMR, including ^1^H–^1^H TOCSY and ^1^H–^13^C HSQC experiments.

Signals corresponding to various free amino acids were observed in the 0.5–3.3 ppm spectral range, including alanine, arginine, asparagine, aspartic acid, glutamine, γ-aminobutyric acid, isoleucine, leucine, and threonine. Organic acids such as acetic acid were also detected in this range, along with other compounds, including choline, dihydrocapsiate, dihydrocapsaicin, and capsaicin. Signals corresponding to α-glucose, β-glucose, fructose, sucrose, and trehalose were observed in the 3.3–5.5 ppm range. The free amino acids detected in this range included homoserine and proline, while malic acid was the predominant organic acid, and capsiate was also identified. The third region between 5.5 and 10.0 ppm contained signals corresponding to the amino acid and phenolic aromatic rings, including those of the histidine, phenylalanine, tryptophan, and tyrosine residues. Nucleotides including cytosine and nicotinamide adenine dinucleotide^+^ were also detected. Notably, the seeds did not contain capsinoids and specific types of capsaicin such as dihydrocapsiate, dihydrocapsaicin, and capsaicin.

### 3.2. Multivariate Statistical Analysis of the ^1^H NMR Spectra

#### 3.2.1. Comparison of the Pericarp, Placenta, and Seed Components

**Figure 2:**
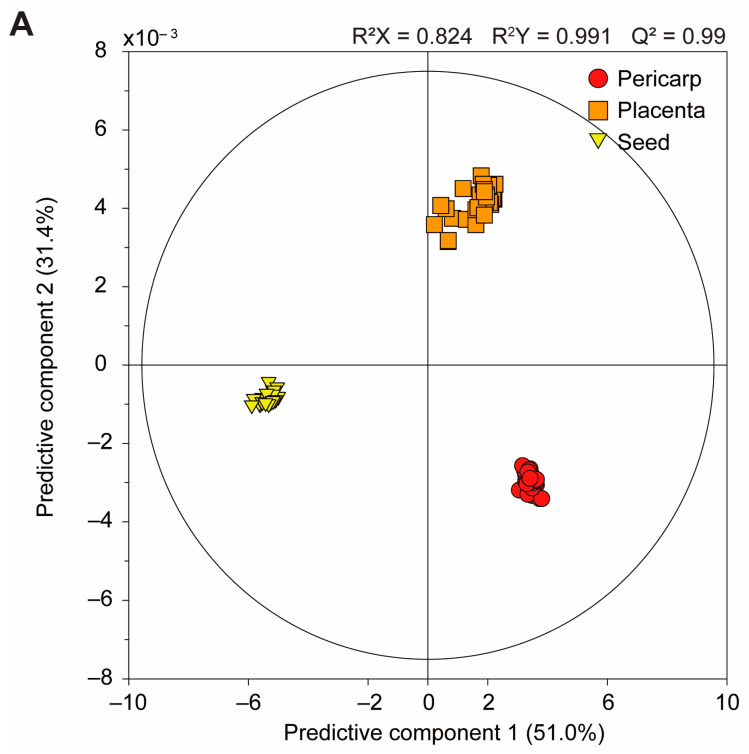

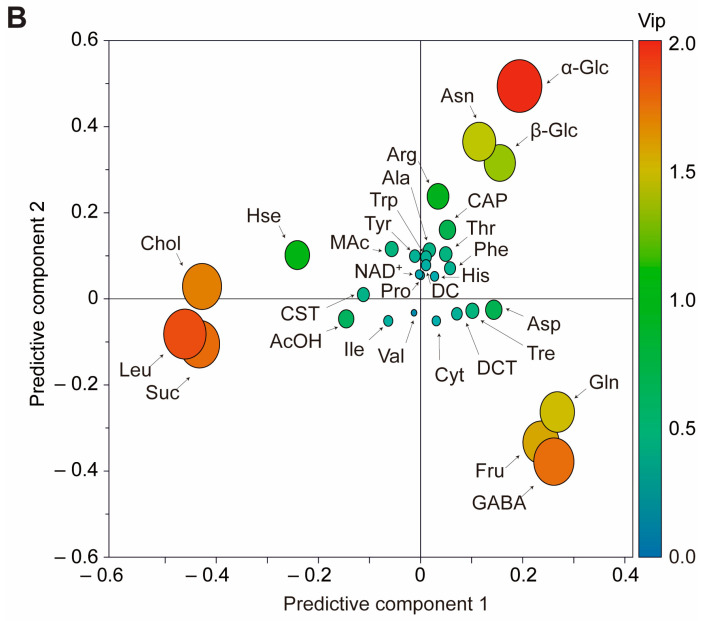
OPLS-DA score plots derived from the ^1^H NMR spectra (800 MHz) of each component in the pericarp, placenta, and seeds of the three cultivars (**A**). Corresponding loading of the scatter plot derived from targeted metabolite profiling (**B**). Thirty samples of each edible part were obtained (pericarp, ●; placenta, ■; seed, ▼), totaling 90 samples (*n* = 30).

Figure 2A illustrates the corresponding clustering of the different plant components, while Figure 2B shows the scatter plot loading. Three primary groups were identified based on two predictive components, namely predictive component one (PC 1; 51%) and PC 2 (31.4%). The OPLS-DA model, which comprised two predictive components, yielded R^2^X, R^2^Y, and Q^2^ values of 0.824, 0.991, and 0.99, respectively, indicating that the OPLS-DA model describes the input data well and accurately predicts the target variable. This suggests that the model has a good fit to the data with a low probability of overfitting. The placenta, pericarp, and seeds are situated in the first, fourth, and third quadrants, respectively, which is a symmetrical point. The pericarp and placenta are positioned on the right side of PC 1, while the seeds are located on the left; consequently, the seeds were the first component to be distinguished. Furthermore, PCA (Figure S1) yields a similar pattern to that found using OPLS-DA. Nevertheless, the OPLS-DA results demonstrate a slightly more pronounced clustering.

Figure 2B shows the variables associated with the similarity/dissimilarity of the pericarp, placenta, and seed observations. Additionally, the variable importance in projection (VIP) values of each variable are indicated by size and color. The VIP values of the compounds were ranked, with α-glucose having the highest value, followed by leucine, sucrose, γ-aminobutyric acid, choline, fructose, glutamine, asparagine, β-glucose, and homoserine. The pericarp components with the highest VIP values were γ-aminobutyric acid, fructose, and glutamine; the corresponding high-VIP components in the placenta were α-glucose, asparagine, and β-glucose; and those in the seeds were leucine, sucrose, choline, and homoserine for the seeds.

#### 3.2.2. Comparison of the Pericarp, Placenta, and Seed Components of the Cultivars

**Figure 3:**
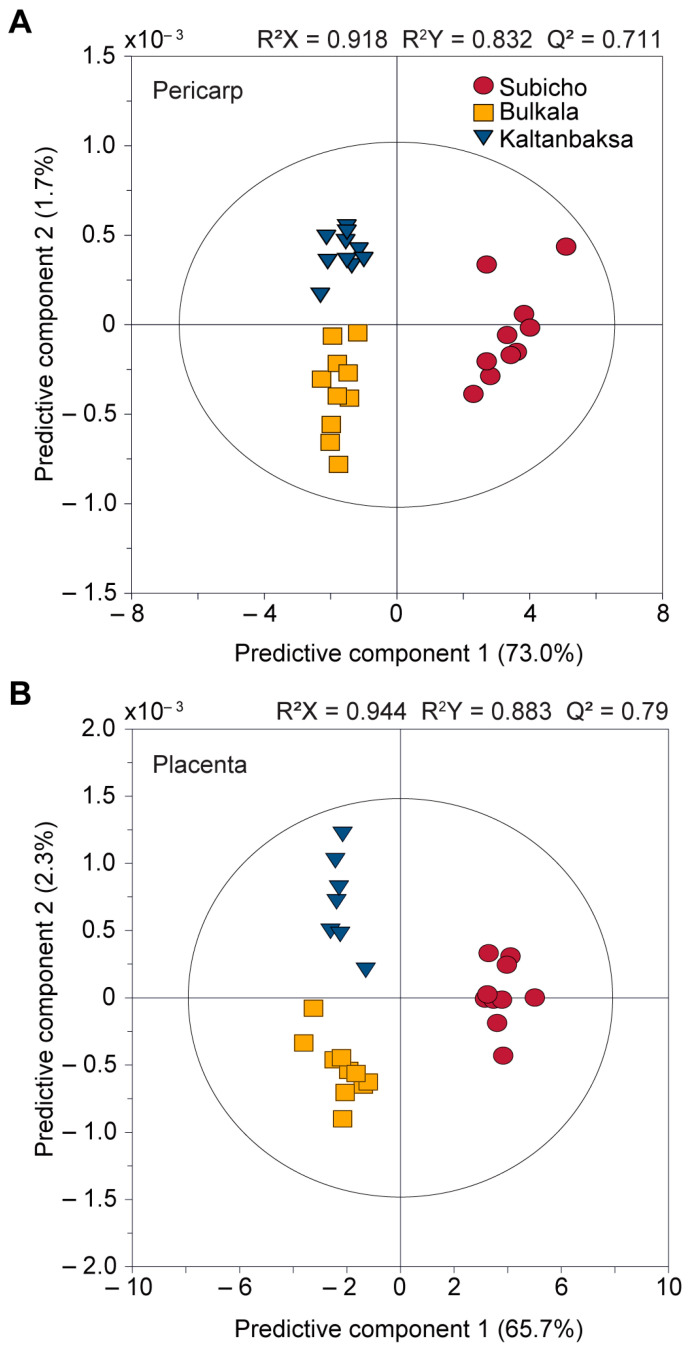

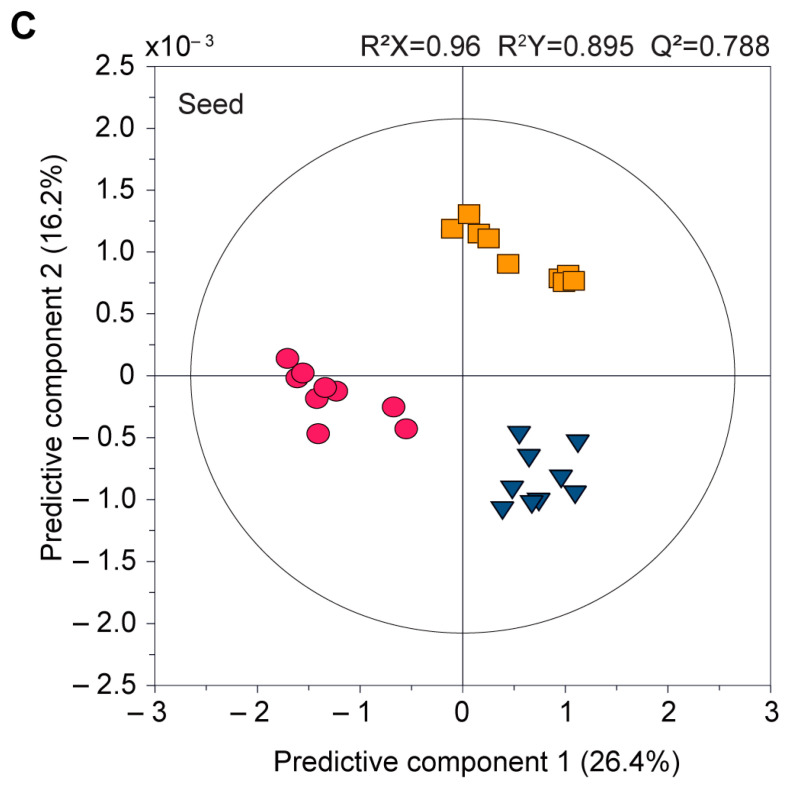
OPLS-DA score plots derived from the ^1^H NMR spectra (800 MHz) of the pericarp (**A**), placenta (**B**), and seed components (**C**) of the three cultivars. Ten samples of the three cultivars (Subicho, ●; Bulkala, ■; Kaltanbaksa, ▼) were obtained, totaling thirty samples (*n* = 10).

The metabolite differences in plant components of the cultivars were determined by OPLS-DA (Figure 3). The OPLS-DA results demonstrated a more distinct separation and stronger clustering among all components (pericarp, placenta, and seeds), thereby highlighting the superior discriminative power of OPLS-DA relative to that of PCA (Figure S2). Moreover, statistical validation of each OPLS-DA model using permutation analysis with 200 model permutations confirmed the validity of the models (Figure S3). The model for evaluating cultivar differences in the pericarp, shown in Figure 3A, comprised two predictive and two orthogonal components. The R^2^X, R^2^Y, and Q^2^ values were 0.918, 0.832, and 0.711, respectively. Upon examination of the predictive components, PC 1 and PC 2 accounted for 73% and 1.7%, respectively. The Subicho cultivar was positioned on the right-hand side of PC 1, whereas the Bulkala and Kaltanbaksa cultivars were positioned on the left, rendering them easily distinguishable. The greatest contributors were γ-aminobutyric acid and aspartic acid.

The model used to evaluate cultivar differences in the placenta, shown in Figure 3B, comprised two predictive and three orthogonal components, wherein the R^2^X, R^2^Y, and Q^2^ values were 0.944, 0.883, and 0.79, respectively. When examining the predictive components, PC 1 and PC 2 accounted for 65.7% and 2.3%, respectively. The Subicho cultivar was located to the right of PC 1, while the Bulkala and Kaltanbaksa cultivars were located to the left. These two cultivars were distinguished first because their characteristics are similar to those of the previous pericarp results. Asparagine, trehalose, and β-glucose had the greatest impact on dividing the PC 1 axis into two large groups.

The model illustrated in Figure 3C was designed to capture cultivar variations in the seeds and incorporated two predictive and four orthogonal components. Although the model exhibited a robust performance with high R^2^X (0.96), R^2^Y (0.895), and Q^2^ (0.788) values, a closer examination of the predictive components raised concerns. The combined explanatory power of PC 1 (26.4%) and PC 2 (16.2%) was ~42.6%, which is below the threshold of 50%. We therefore considered that the seed model does not fully explain the seed metabolite characteristics. Furthermore, varietal differences in the seeds were not as clearly divided along the predictive component axis as those in the pericarp and the placenta. The key metabolites that distinguished PC 1 were sucrose, asparagine, and leucine.

#### 3.2.3. Comparison of the Landrace and Disease-Resistant Cultivars Based on the Pericarp and Placenta Components

**Figure 4:**
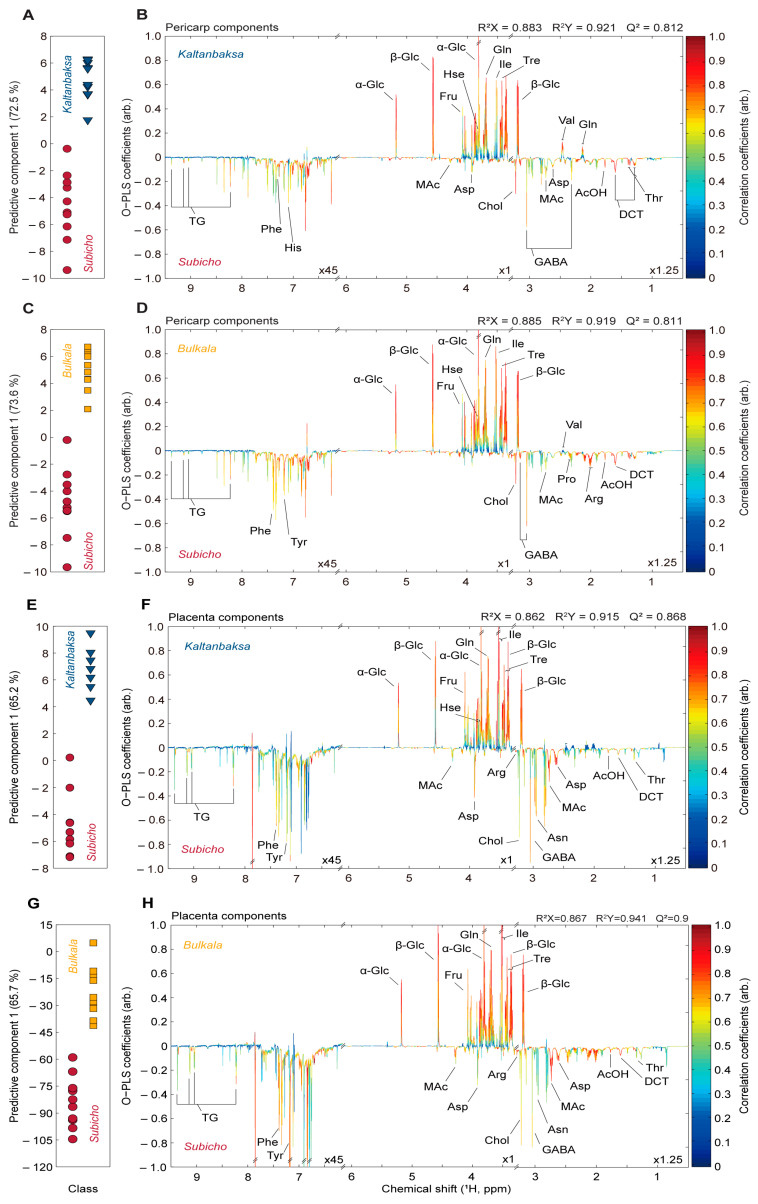
OPLS-DA scores (**A**,**C**,**E**,**G**) and coefficient or loading plots (**B**,**D**,**F**,**H**) for metabolic differentiation between the Subicho and Kaltanbaksa pericarp components (**A**,**B**), Subicho and Bulkala pericarp components (**C**,**D**), Subicho and Kaltanbaksa placenta components (**E**,**F**), and Subicho and Bulkala placenta components (**G**,**H**) for identification of the chili pepper metabolites responsible for metabolic differentiation. The colors in the loading plot correspond to the correlation among variables. All OPLS-DA models were generated using one predictive and one orthogonal component. Their reliabilities and predictabilities are indicated by R^2^X, R^2^Y, and Q^2^. Abbreviations of the names of the assigned metabolites are listed in Table S1. Ten samples were obtained from the edible parts of each cultivar (*n* = 10).

The ^1^H NMR spectra of the chili pepper specimens were used to generate OPLS-DA scores and loading plots to identify the metabolites responsible for the differences between the cultivars. Statistical analyses of extracts from the pericarp and placenta components allowed for pairwise comparisons between the Subicho and Kaltanbaksa cultivars and between the Subicho and Bulkala cultivars (Figure 4). Varying levels of particular compounds were observed in the pericarp of the Subicho and Kaltanbaksa cultivars. The Kaltanbaksa cultivar exhibited higher levels of glucose, fructose, homoserine, glutamine, isoleucine, trehalose, and valine than the Subicho cultivar, as well as lower levels of trigonelline, phenylalanine, histidine, malic acid, aspartic acid, choline, γ-aminobutyric acid, acetic acid, dihydrocapsiate, and threonine (Figure 4B). A comparison of the pericarp metabolites of the Subicho and Bulkala cultivars showed that the Bulkala cultivar possessed higher levels of glucose, fructose, homoserine, glutamine, isoleucine, trehalose, and valine than the Subicho cultivar. In contrast, the Bulkala cultivar contained lower levels of trigonelline, phenylalanine, tyrosine, choline, γ-aminobutyric acid, malic acid, proline, arginine, acetic acid, and dihydrocapsiate (Figure 4D). The metabolites present in the placentas of the Subicho and Kaltanbaksa cultivars were attributed to higher levels of glucose, fructose, homoserine, glutamine, isoleucine, and trehalose in the latter, as well as lower levels of trigonelline, phenylalanine, tyrosine, malic acid, aspartic acid, arginine, choline, γ-aminobutyric acid, asparagine, acetic acid, dihydrocapsiate, and threonine (Figure 4F). Moreover, the placenta from the Kaltanbaksa cultivar exhibited lower levels of most metabolites than that from the Subicho cultivar, including trigonelline, phenylalanine, tyrosine, malic acid, aspartic acid, arginine, choline, γ-aminobutyric acid, asparagine, acetic acid, dihydrocapsiate, and threonine; however, the levels of glucose, fructose, isoleucine, and trehalose were significantly higher (Figure 4H).

### 3.3. Relative Amounts of Different Metabolites Based on ^1^H NMR Spectroscopy

#### 3.3.1. Comparison of the Pericarp, Placenta, and Seed Components

**Figure 5:**
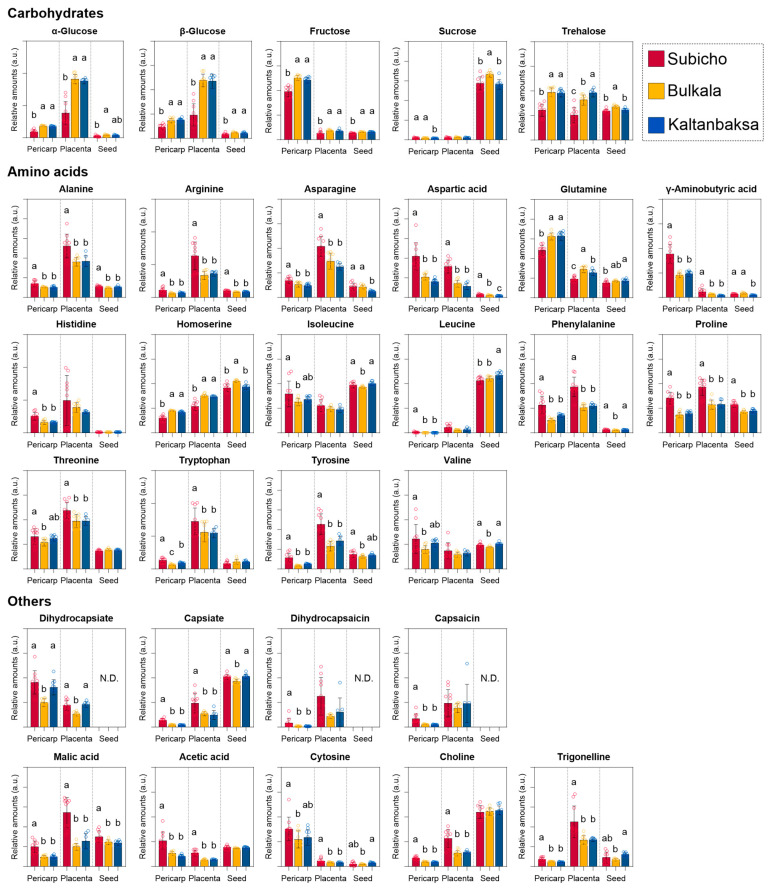
Quantification of individual metabolites in capsicum and comparison of the relative metabolite contents of the pericarp, placenta, and seed components of the three cultivars. Vertical bars denoted by different lowercase letters indicate significant differences between compounds at *p* < 0.05, as defined by Duncan’s multiple range test. Ten samples were obtained from the edible parts of each cultivar (*n* = 10). N.D. denotes values that were not detected. Different colored circles represent individual data points for each sample.

Figure 5 shows the relative amounts of different metabolites in the pericarp, placenta, and seed components of the three cultivars, as calculated from the integrals of the ^1^H NMR signals. The differences in the metabolite contents of the pericarp, placenta, and seed components of the chili peppers were attributed to the different functions of these structures in fruit development and functionality [19]. The pericarp serves as the outer integument of the chili pepper, shielding it from environmental stressors such as pathogens, UV radiation, and physical attack. Consequently, the antioxidants and other metabolites found in the pericarp actively participate in defense mechanisms and thus play significant roles in fruit fortification [20]. In the present study, statistical analysis of the fructose, γ-aminobutyric acid (GABA), glutamine, and aspartic acid contents of the pericarp revealed their potential key roles in this component. Indeed, the sugar contents of twenty-one cultivars from four different species were examined, and it has been reported that the pericarp contains more sugars than the placenta [21]. Additionally, the GABA content is known to be higher in the pericarp than in the placenta and to correlate strongly with the total amino acid levels [22]. Snowden et al. [23] suggested that GABA, glutamine, and aspartic acid levels significantly influence the taste and nutritional quality of the ripening red tomato pericarp. GABA levels peaked when the fruit reached its maximum volume and declined as the fruit ripened, while glutamine and aspartic acid also accumulated. These findings suggest that metabolic activity occurs in the pericarp during ripening. The results obtained herein therefore support the hypothesis that the pericarp undergoes significant metabolic changes during ripening, particularly in the accumulation of sugars and amino acids, enhancing its influence on the overall taste and nutritional profile of the fruit.

The placenta connects the seeds to the fruit and plays a crucial role in transporting nutrients and supporting seed development. The metabolites present in the placenta are therefore involved in nutrient storage, transport, and signaling to support seed development [24]. We found that the placenta contained higher glucose, asparagine, and arginine contents than the other parts of the chili pepper. Additionally, high levels of capsaicin, which is responsible for the spicy and pungent flavor of the chili [25], were detected. Indeed, capsaicin and its related compounds are typically distributed throughout the placenta, accounting for 85–95% of the total capsaicinoid content of chili peppers. The sum of the capsaicin and dihydrocapsaicin contents of three varieties of red pepper (*Capsicum annuum* L.) exhibited considerable variation, ranging from 0.79 to 58.31 μg/g FW in the pericarp and from 1.20 to 1035.70 μg/g FW in the placenta [26]. Notably, capsaicin and dihydrocapsaicin, which are found in the pericarp and placenta, serve protective functions in plants; however, their concentrations are lower in the seeds because they can inhibit germination [27].

Chili pepper seeds are well-known reproductive structures that act as reservoirs of essential nutrients and critical bioactive compounds, including sterols, triterpenes, organic acids, fatty acids, and volatile compounds [28]. In the current study, leucine, sucrose, choline, and homoserine were detected in the seed components of the various chili peppers. Together with the branched-chain amino acids isoleucine and valine, the highly abundant leucine influences numerous metabolic pathways, including plant growth, defense, and the production of flavor compounds [29]. In addition, the seed components contained higher levels of sucrose than glucose and fructose. Sucrose is essential to meet the energy requirements of plant embryos during germination [30]. Furthermore, choline, a common plant growth regulator that plays an important role in germination by accelerating cell division and growth, was also detected in abundance in the seed specimens [31]. 

The key compounds and metabolic characteristics associated with the different parts of the chili pepper plant are attributed to their specific functions. The results obtained herein will therefore be useful in the context of food science and technology.

#### 3.3.2. Comparison of the Components in the Pericarp and Placenta of the Cultivars

The carbohydrate and amino acid metabolomic characteristics of the conventional Subicho cultivar, which has a long history of cultivation in Korea, were compared with those of the recently bred Bulkala and Kaltanbaksa cultivars, which exhibit multi-disease resistance characteristics. In the current study, higher levels of specific carbohydrates, including sucrose and hexoses, accumulated in the multi-disease-resistant cultivars relative to the conventional cultivar. In this context, Bolton [32] suggested that the accumulation of soluble carbohydrates may trigger a defense response by suppressing photosynthetic genes. 

Soluble sugars such as sucrose and hexoses are known to enhance stress tolerance by suppressing stress-related genes and promoting the expression of growth-related genes [33]. These carbohydrates have crucial physiological functions as energy sources and signaling molecules that regulate growth and developmental processes. Furthermore, the resistant phenotypes that overexpress genes associated with carbohydrate metabolism in transgenic plants provide convincing evidence for the role of carbohydrates in defense responses. According to the ‘sugar signaling’ hypothesis, which is supported by the fact that sugars can act as signaling molecules that induce defense genes [34], changes in sugar levels at an infection site trigger signaling cascades that activate the salicylic acid pathway and upregulate defense genes, leading to physiological changes that repel pathogens [35]. Collectively, the transcriptomic and genetic data support the general view that avirulent pathogens or pathogen-derived elicitors induce the expression of genes involved in the carbohydrate metabolic processes, including glycolysis, the pentose phosphate pathway, and the tricarboxylic acid cycle [36]. The expression of these genes affects defense responses, including the generation of reactive oxygen species and the activation of pathogenesis-related genes that precede the onset of the hypersensitive response [37]. Accordingly, it is widely accepted that the accumulation of carbohydrate metabolites is a common mechanism that reliably avoids the negative effects of stress. However, carbohydrate accumulation is dependent on the type of stress to which the plant is exposed [38].

Distinct metabolic pathways involving amino acids are integral components of the plant immune system. To date, various studies genetically manipulated amino acid metabolism to improve disease resistance in plants. Liu et al. [39] artificially reduced glutamine, alanine, and proline levels in *Arabidopsis thaliana* by mutating lysine histidine transporter 1 (LHT1) to enhance the plant’s resistance to bacterial, fungal, and oomycete pathogens. Additionally, the LHT1 mutant exhibited heightened levels of callose deposition, increased accumulation of salicylic acid, and constitutive expression of pathogenesis-related protein 1 [40]. Amino acids such as glutamine, alanine, and proline are essential for protein synthesis, nitrogen transport, and osmotic regulation, playing vital roles in plant growth and development. Moreover, the proline metabolism integrates regulation of the redox and energy balance, growth, and defense processes and is coordinated with numerous pathways in various cellular compartments. Indeed, Fabro et al. [41] reported that certain resistant plants may contain low proline levels. A mutant of the proline metabolism-related gene proline dehydrogenase (ProDH) in *Arabidopsis thaliana* demonstrates a high resistance to avirulent pathogens. ProDH catalyzes the conversion of proline to pyrroline-5-carboxylate; thus, a ProDH mutant exhibits limited levels of proline accumulation. In another study, Alvarez et al. [42] reported that proline is primarily involved in resistance-related processes, including hypersensitive responses and effector-triggered immunity, and in the plant’s defense against pathogens. The accumulation of proline in plants typically adversely affects plant growth and induces cell death under non-stress conditions, whereas low concentrations reduce cell death [43]. In non-stressed plants, proline acts as a signaling molecule; however, plants infected with pathogens exhibit increased proline levels, serving as a positive regulator of the immune response [44]. In the absence of stress, the pericarp and placenta of the disease-resistant cultivars contained lower alanine and proline levels than those of the Subicho cultivar. Moreover, the resistant cultivars exhibited significantly lower levels of aspartate, threonine, and tryptophan. Similarly, reduced levels of aspartate, glutamine, threonine, and tryptophan, along with increased levels of jasmonic acid, were observed in tomatoes treated with β-aminobutyric acid (BABA), thereby indicating the role of jasmonic acid in controlling the plant’s defense response [45]. BABA is known to produce an endogenous resistance-inducing stress metabolite that enhances disease resistance in various crops, including common beans, potatoes, grapes, tomatoes, peppers, cabbage, and fruits [46]. These amino acids play critical roles in metabolic pathways that determine the plant’s overall stress response and immune function. Based on the findings of the present study, it is evident that changes in amino acid levels in pepper cultivars, particularly reductions in the aspartate, threonine, and tryptophan levels, are associated with stress responses. This correlation underscores the importance of these amino acids in the adaptation and defense mechanisms of plants under biotic conditions, as well as in maintaining a balance between growth and defense.

## 4. Conclusions

A comparison and statistical analysis of the metabolic composition of different plant components (pericarp, placenta, and seeds) of conventional landrace and disease-resistant chili pepper cultivars is presented. This study achieved its aim of improving our understanding of the impact of various metabolites on various characteristics and attributes of chili peppers. The metabolite composition in different parts of the chili pepper cultivars varied significantly owing to their respective physiological functions. Furthermore, the carbohydrate and amino acid metabolisms of the pericarp and placenta of the disease-resistant cultivars differed significantly from those of the same components of the conventional landrace cultivar, indicating that resistance was achieved through complex interactions. In particular, the accumulation of specific carbohydrates such as sucrose and hexoses improves stress tolerance and activates defense pathways. Similarly, the reduction in the levels of certain amino acids, such as alanine, proline, threonine, and tryptophan, reallocates resources to bolster defense mechanisms at the expense of growth under stress conditions. These complex interactions between metabolic pathways demonstrate the multifaceted nature of disease resistance in chili peppers. Metabolomics is therefore an invaluable tool in elucidating the mechanisms of resistance in the food and agronomic industries. Chili peppers belong to the Solanaceae family and therefore provide a valuable model for studying the metabolisms of other commercially important crops, including tobacco, potatoes, tomatoes, and eggplant. Further investigations will aim to identify the key metabolites associated with taste, flavor, and disease characteristics in chili peppers grown in different regions.

## Data Availability

The original contributions presented in the study are included in the article/Supplementary Material, further inquiries can be directed to the corresponding author.

## Supplementary Materials

The following supporting information can be downloaded at: https://www.mdpi.com/article/10.3390/foods13131966/s1, Figure S1: PCA score plots derived from the ^1^H NMR spectra (800 MHz) of the pericarp, placenta, and seed components of the three cultivars; Figure S2: PCA score plots derived from the ^1^H NMR spectra (800 MHz) of the pericarp (A), placenta (B), and seed components (C) of the three cultivars; Figure S3: Permutation analysis validates the discrimination of pericarp (Subicho; A, Bulkala; B, Kaltanbaksa; C), placenta (Subicho; D, Bulkala; E, Kaltanbaksa; F), and seed (Subicho; G, Bulkala; H, Kaltanbaksa; I) by the OPLS-DA model in Figure 3; Figure S4: Total correlation spectrum of the chili pepper specimen; Figure S5: ^1^H-^13^C heteronuclear single-quantum correlation spectrum of the chili pepper specimen; Table S1: Metabolites identified in the chili pepper cultivars.
