# Supplementary material for: Comparative 1H NMR-Based Metabolomics of Traditional Landrace and Disease-Resistant Chili Peppers (Capsicum annuum L.)
Source: Foods. 2024 Jun 21;13(13):1966. doi: 10.3390/foods13131966 (PMC11241277; doi:10.3390/foods13131966)
Supplement: Supplementary file 1 [file foods-13-01966-s001.zip › foods-3053222-supplementary.pdf]

Table S1. Metabolites identified in the chili pepper cultivars

Abbreviation	Metabolite	δ¹H (ppm)	δ¹³C (ppm)	Shape
α–Glc	α–Glucose	5.18	95.16	d
		3.82	55.44	OVLP
		3.79	84.14	OVLP
β–Glc	β–Glucose	4.57	99.23	d
		3.70	76.03	OVLP
Fru	Fructose	4.08	77.77	dd(OVLP)
Suc	Sucrose	5.41	95.05	d
		4.16	79.88	d
		4.03	76.07	s(OVLP)
Tre	Trehalose	3.88	63.95	m
Ala	Alanine	1.50	18.97	d
		3.70	53.40	q(OVLP)
Arg	Arginine	3.24	43.41	t(OVLP)
		1.92	30.91	m
Asn	Asparagine	3.92	54.31	q(OVLP)
		2.96	37.23	dd
Asp	Aspartic acid	3.92	54.29	m(OVLP)
		2.81	39.25	d(OVLP)
		2.62	39.26	d
Gln	Glutamine	2.47	33.88	m
		2.14	29.23	m
		3.70	57.10	t(OVLP)
GABA	γ–Aminobutyric acid	3.01	42.21	t
		2.32	37.44	t(OVLP)
		1.91	26.46	M
His	Histidine	7.10	119.61	s
Hse	Homoserine	3.83	55.39	dd(OVLP)
Ile	Isoleucine	3.53	63.23	d(OVLP)
		1.02	19.40	d
Leu	Leucine	3.70	57.10	t(OVLP)
		1.49	24.42	m(OVLP)

Phe	Phenylalanine	7.34	132.09	m(OVLP)
		7.33	130.26	m(OVLP)
		7.39	131.76	t
Pro	Proline	4.12	61.24	dd
Thr	Threonine	4.21	68.76	d
		3.48	63.44	d(OVLP)
		1.34	22.51	m
Trp	Tryptophan	7.72	121.06	d
		7.45	114.29	d
Tyr	Tyrosine	7.18	133.40	ddd
Val	Valine	3.70	64.95	d
		2.46	17.38	d(OVLP)
		1.04	17.38	m(OVLP)
MAc	Malic acid	4.29	72.48	dd(OVLP)
		2.74	47.45	dd(OVLP)
AcOH	Acetic acid	1.77	23.47	s(OVLP)
Cyt	Cytosine	5.82	98.24	d
Chol	Choline	3.22	56.59	s
		4.00	63.20	m(OVLP)
TG	Trigonelline	9.37	145.46	s
		9.13	145.47	d
		9.04	150.31	m
		8.23	130.44	m
DCT	Dihydrocapsiate	2.36	–	t(OVLP)
CST	Capsiate	5.05	24.73	s(OVLP)
DC	Dihydrocapsaicin	0.86	24.68	d(OVLP)
CAP	Capsaicin	0.95	18.81	d
		0.85	24.71	d(OVLP)

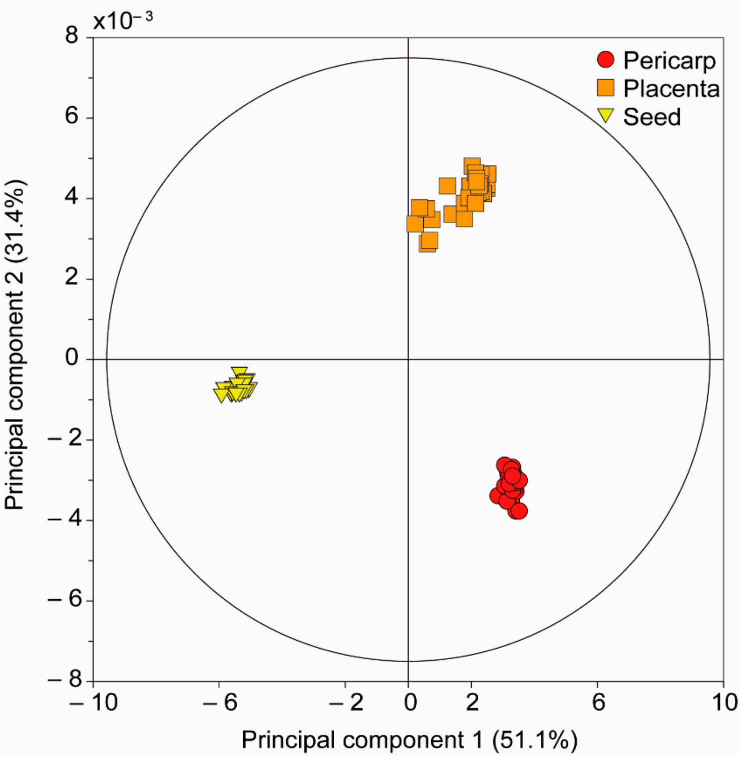

Figure S1. PCA score plots derived from the ^{1}H NMR spectra (800 MHz) of the pericarp, placenta, and seed components of the three cultivars. Thirty samples of each edible part were obtained (pericarp, ●; placenta, ■; seed, ▼), totaling 90 samples (n=30).

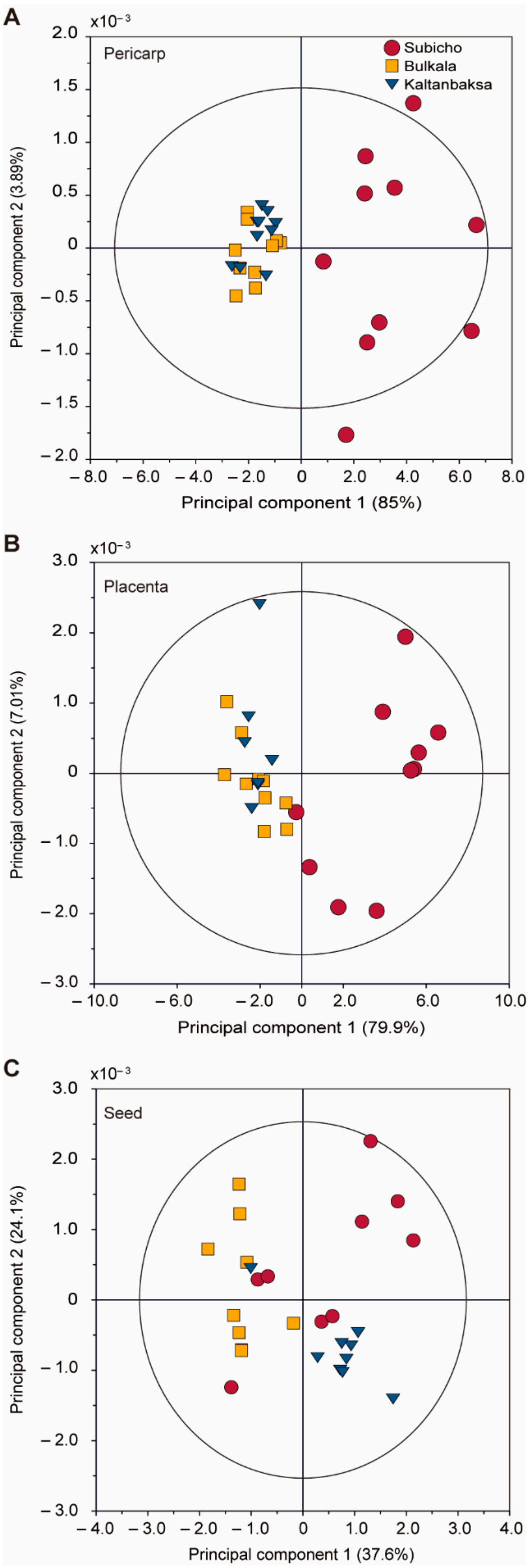

Figure S2. PCA score plots derived from the ^{1}H NMR spectra (800 MHz) of the pericarp (A), placenta (B), and seed components (C) of the three cultivars. Ten samples of the three cultivars (Subicho, ●; Bulkala, ■; Kaltanbaksa, ▼) were obtained, totaling thirty samples (n=10).

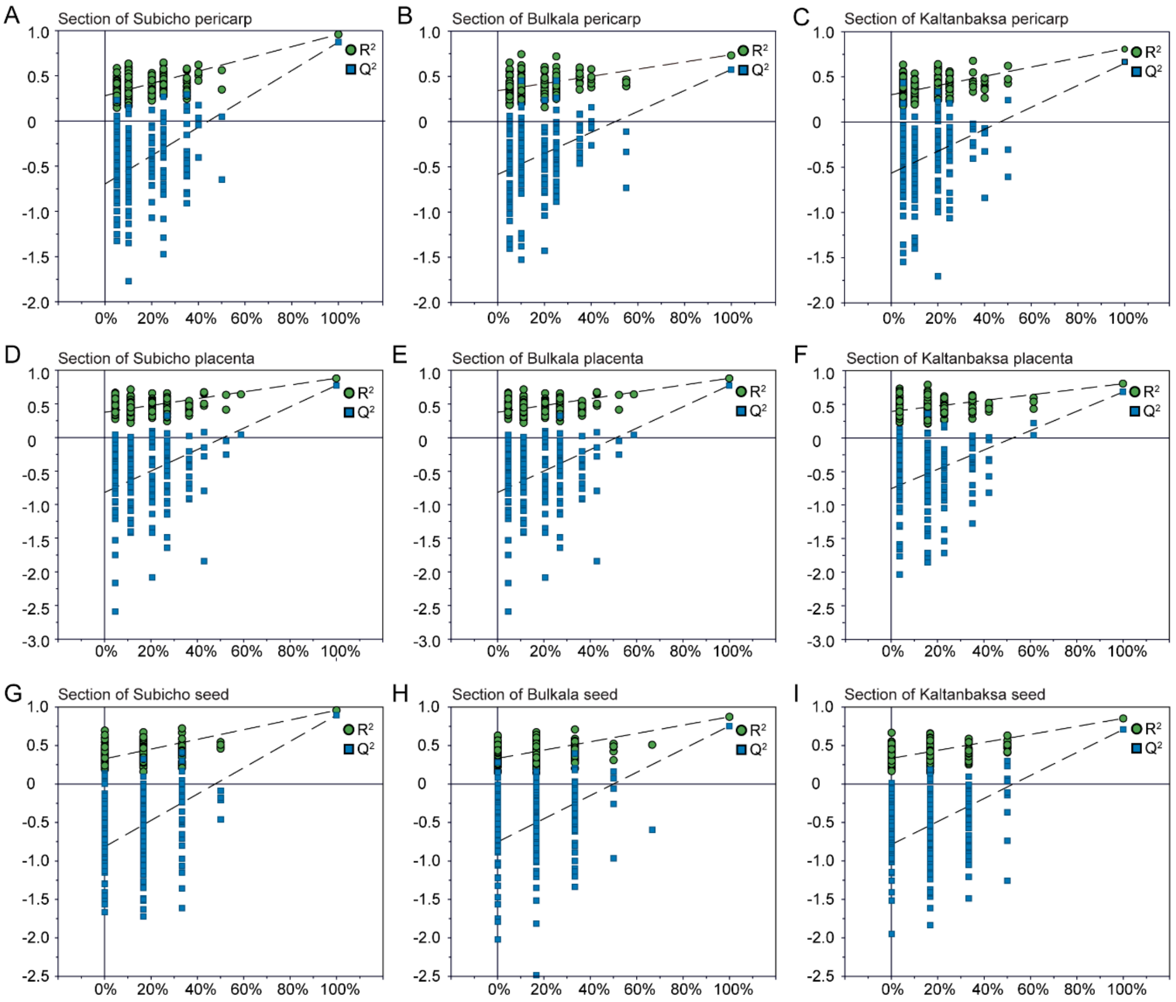

Figure S3. Permutation analysis validates discrimination of pericarp (Subicho; A, Bulkala; B, Kaltanbaksa; C), placenta (Subicho; D, Bulkala; E, Kaltanbaksa; F), and seed (Subicho; G, Bulkala; H, Kaltanbaksa; I) by the OPLS-DA model in Figure 3.

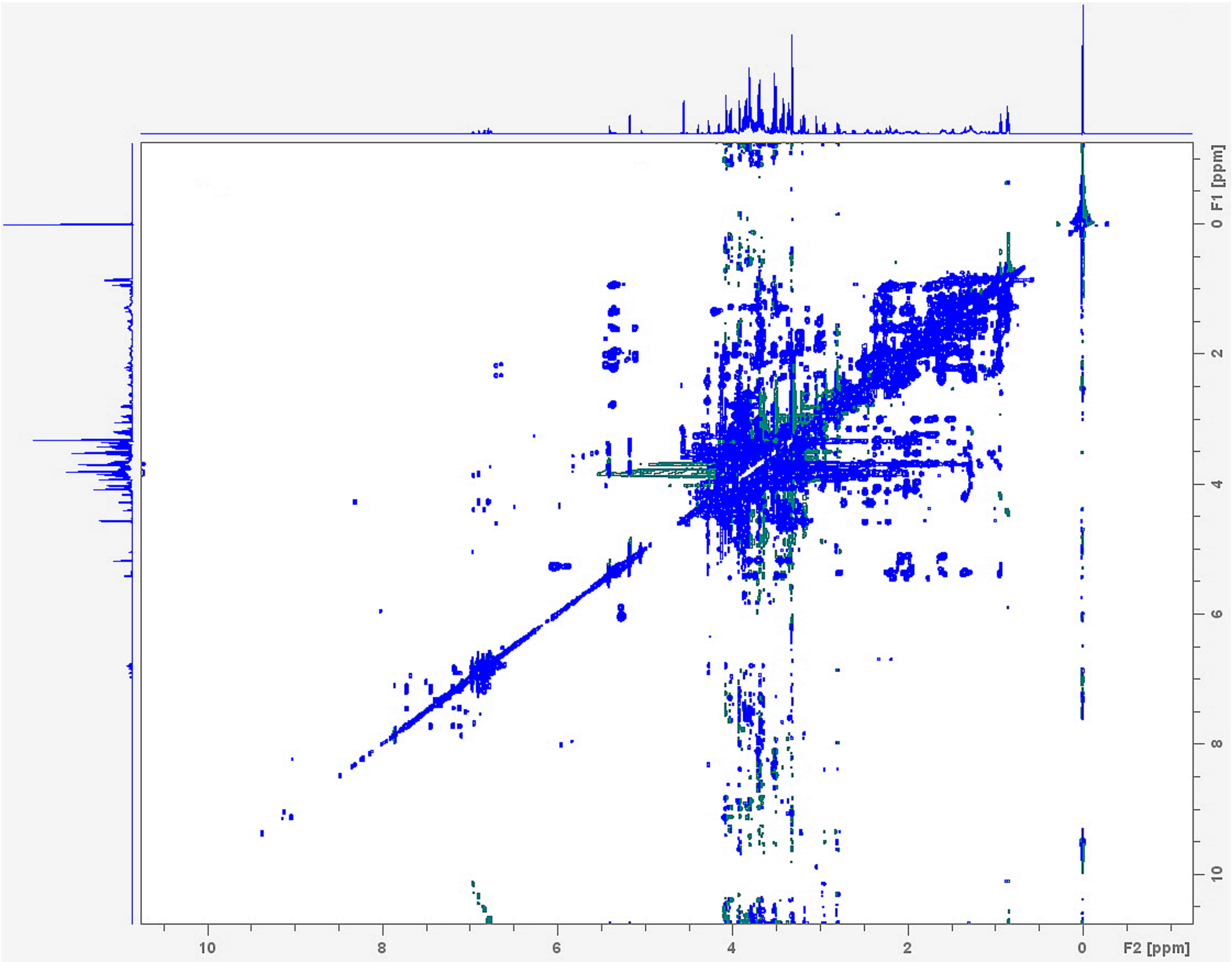

Figure S4. Total correlation spectrum of the chili pepper specimen.

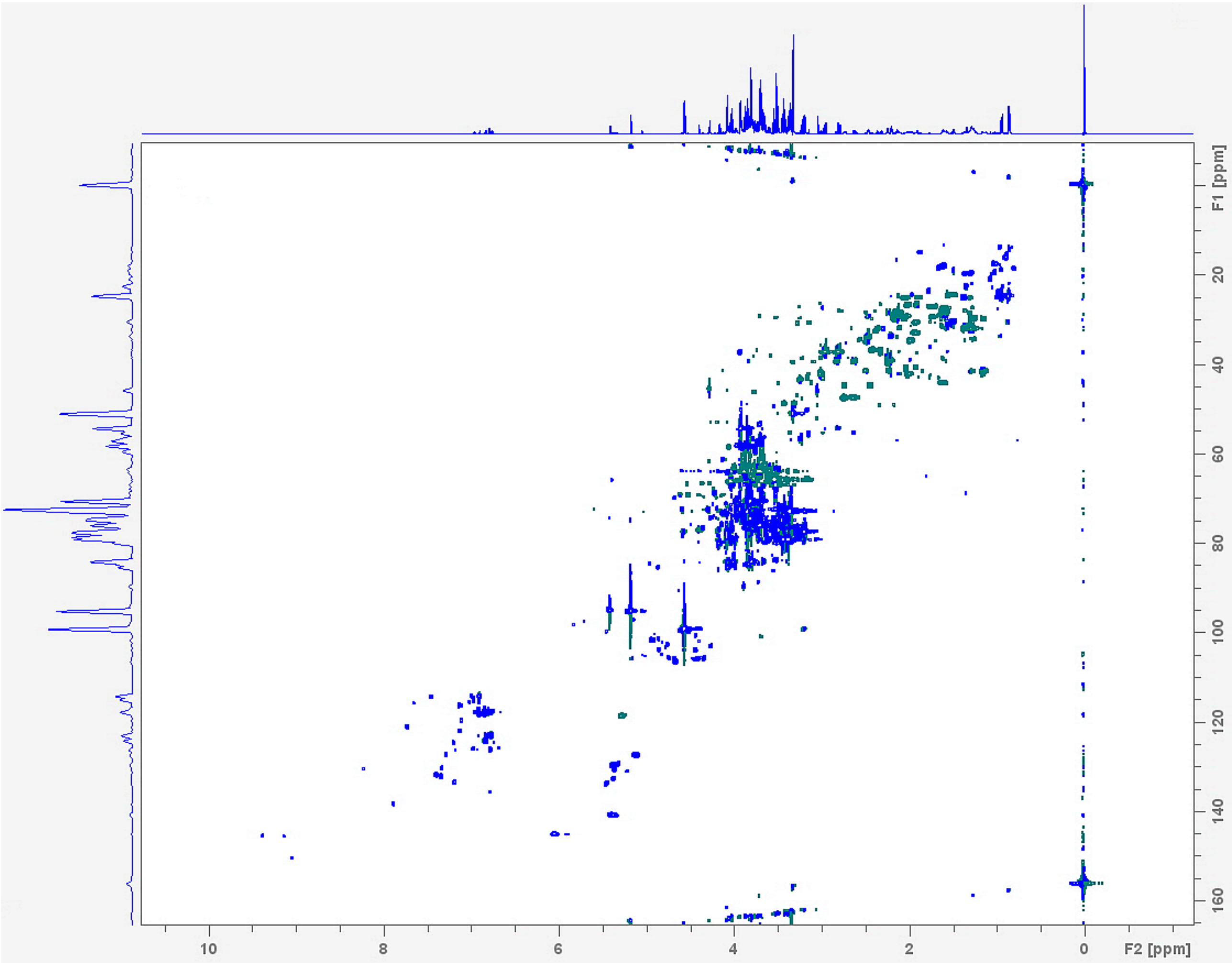

Figure S5. ^{1}H-^{13}C heteronuclear single-quantum correlation spectrum of the chili pepper specimen.
